# Advancing Barrett’s Esophagus Segmentation: A Deep-Learning Ensemble Approach with Data Augmentation and Model Collaboration

**DOI:** 10.3390/bioengineering11010047

**Published:** 2024-01-02

**Authors:** Jiann-Der Lee, Chih Mao Tsai

**Affiliations:** 1 Department of Electrical Engineering, Chang Gung University, Taoyuan 33302, Taiwan; 2 Department of Neurosurgery, Chang Gung Memorial Hospital at Linkou, Taoyuan 33305, Taiwan; 3 Department of Electrical Engineering, Ming Chi University of Technology, New Taipei City 24330, Taiwan

**Keywords:** Barrett’s esophagus, deep learning, medical segmentation, U-Net, Deeplabv3+, data augmentation, ensemble

## Abstract

This approach provides a thorough investigation of Barrett’s esophagus segmentation using deep-learning methods. This study explores various U-Net model variants with different backbone architectures, focusing on how the choice of backbone influences segmentation accuracy. By employing rigorous data augmentation techniques and ensemble strategies, the goal is to achieve precise and robust segmentation results. Key findings include the superiority of DenseNet backbones, the importance of tailored data augmentation, and the adaptability of training U-Net models from scratch. Ensemble methods are shown to enhance segmentation accuracy, and a grid search is used to fine-tune ensemble weights. A comprehensive comparison with the popular Deeplabv3+ architecture emphasizes the role of dataset characteristics. Insights into training saturation help optimize resource utilization, and efficient ensembles consistently achieve high mean intersection over union (IoU) scores, approaching 0.94. This research marks a significant advancement in Barrett’s esophagus segmentation.

## 1. Introduction

Barrett’s esophagus (BE) is a condition that affects the lining of the esophagus and increases the risk of developing esophageal cancer. Early detection of neoplasia in BE is crucial for effective treatment and better prognosis. However, the heterogeneity of BE poses a challenge for exact diagnosis. In this study, we used public data from Kaggle’s HyperKvasir dataset [1], which supplies a comprehensive collection of multi-class images and videos for gastrointestinal endoscopy. Nonetheless, it is worth noting that this dataset consisted of only ninety-four images depicting BE, which may be deemed inadequate for effectively training deep-learning models. Considering this limitation, we conducted a comprehensive review of the existing literature and ascertained that the detection of early neoplasia in Barrett’s esophagus ranks as a paramount research priority [2]. Studies have been conducted to find neoplasia in patients with BE, including de Groof et al. [3] and Hashimoto R et al. [4]. However, these studies focused on the early detection of cancer change or dysplasia in severe Barrett’s esophagus. The standard diagnosis of Barrett’s esophagus typically involves a combination of clinical evaluation, endoscopy, and biopsy. Barrett’s esophagus with no dysplasia can be reversed or prevented from deterioration to cancer via early detection and just early conservative treatment deployed.

The U-Net architecture has shown excellent performance in biomedical image segmentation tasks [5]. Its distinctive U-shaped design, including an encoder and decoder pathway, enables the model to capture both local details and global context, making it well suited for segmentation tasks, including BE change. The U-Net model can be considered the state of the art for medical image segmentation tasks. One of the reasons for the wide popularity of U-Net in medical segmentation is its ability to manage less training data. Medical datasets are often small, and the U-Net’s architecture, with or without a pretrained backbone, allows for effective training even with scarce data. Additionally, U-Net has shown robust performance across different medical imaging modalities, such as MRI, CT scans, endoscopy, and microscopy images [6,7]. Numerous variants and extensions of the U-Net model have been proposed to enhance its performance and address specific challenges. For example, U-Net has been widely adopted in various medical imaging applications, including cell segmentation, organ segmentation, and tumor detection. Pan et al. [8] applied fully convolutional neural networks [9] to conduct Barrett’s esophagus segmentation. They focused on the gastroesophageal junction(GEJ) and the squamous-columnar junction(SCJ), respectively. Their study values of the IOU were 0.56 (GEJ) and 0.82 (SCJ), respectively. We aim to expand Barrett’s esophagus segmentation from severe esophageal cancer to Barrett’s esophagus segmentation and focus on segmentation of the isolated esophageal lesion involved with a free excellent APEER annotation tool. Our study is also in line with early diagnosis and treatment of preventive medicine. Considering the adaptable nature of input size, our approach is initiated by a platform of robust segmentation architectures, incorporating pre-trained models from the keras-unet-collection by Sha, Yingkai [10].

## 2. Materials and Methods

### 2.1. Raw Images and the Annotation Method

This study commenced with a dataset comprising 94 images obtained from Kaggle open data, encompassing both short-segment and long-segment Barrett’s esophagus lesions within Barrett’s esophagus cases. The raw images were diverse in shape, size, and resolution.

**Figure 1:**
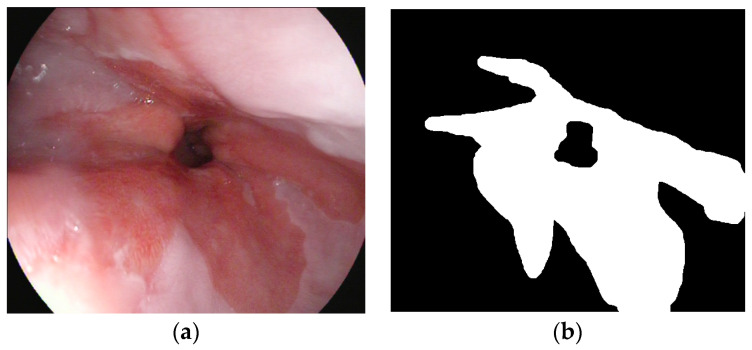
(**a**) An example image and (**b**) its corresponding annotated mask.

We annotated the images using the free APEER annotation tool that is now ZEISS arivis Cloud. In this binary segmentation task, Barrett’s lesion in each image was annotated as foreground (ground truth). Other areas, including gastric folds, were annotated as background. The resultant images and corresponding annotated masks were further sent to 2-step data augmentation. An example image with its corresponding annotated mask is shown in Figure 1.

### 2.2. Data Augmentation

#### 2.2.1. Step 1: Data Augmentation to Create Dataset-192

To enhance dataset diversity and scale, we meticulously applied a series of data augmentation techniques. These techniques included random cropping, Gaussian blur, and the introduction of random noise, orchestrated to imbue the dataset with more richness and variability. Under the principle of maintaining the major features of the images, the first stride involved resizing the images to a size of 512 × 512 pixels, ensuring uniformity in later processing. To introduce controlled variability, we incorporated a crop percentage parameter set at 10%. This parameter dictated that 2.5% of the image dimensions would be symmetrically cropped from all sides, ensuring even distribution across the images and their associated masks. To further amplify diversity and intricacy within the training set, we introduced a Gaussian noise transformation. This augmentation was achieved using the PyTorch library’s transforms.Compose function, executed in a two-step sequence. The first step involved Gaussian blurring using a kernel size of 5, followed by the subsequent introduction of Gaussian noise. Gaussian noise was incorporated using a lambda function, involving the generation of random values from a standard normal distribution, which was then multiplied by a designated standard deviation of 0.5. These randomized values were then added to individual pixels throughout the input image. Through the procedures, our initial dataset, referred to as Dataset-192, was assembled. This dataset consisted of 192 images, which included the original 94 images, augmented by an additional 46 images obtained via cropping, and 52 images generated by applying Gaussian blur and introducing Gaussian noise in a random manner.

#### 2.2.2. Step 2: Data Augmentation to Create Dataset-360

**Figure 2:**
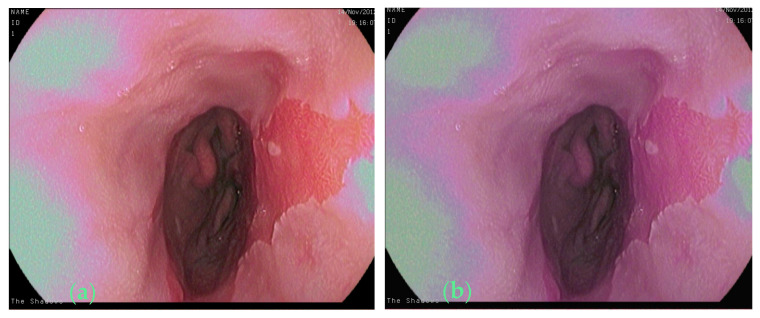
(**a**) Original image, (**b**) the image after color jittering transformation.

**Figure 3:**
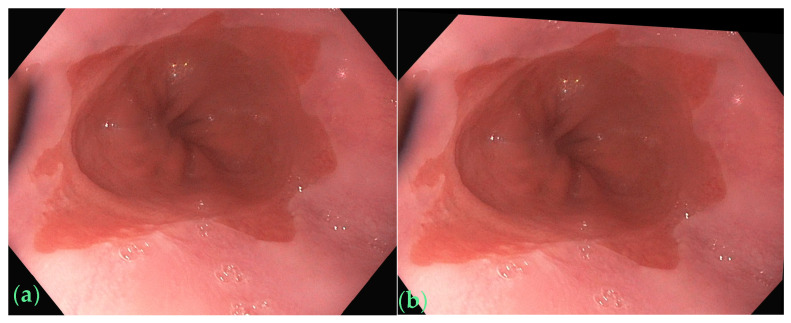
(**a**) Original image, (**b**) the image after affine transformation.

With this refined augmentation approach, our method aimed to capture the intricacies of Barrett’s esophagus images, improve model robustness, and enhance the segmentation performance. We defined the color jittering transformation as follows: brightness = 0.1, contrast = 0.1, saturation = 0.1, hue = 0.1. For affine transformation, we set the angle range between −5 and 5 degrees, the range of scaling factors between 0.95 and 1.05, and the range of translations between −0.01 and 0.01. We implemented a randomized approach for selecting transformation parameters during data augmentation. This approach involved randomly generating values for rotation angle, scaling factor, horizontal translation, and vertical translation. The use of random values within specified ranges for these parameters added variability to the data augmentation. Only one image (about 1%) in the original dataset showed colorful afterimages, as shown in Figure 2a. Figure 2b shows the image after color jittering transformation. We demonstrate an image of the affine transformation in Figure 3b from the original image shown in Figure 3a. By integrating additional augmentation techniques, which involved incorporating 38 images from affine transformation, 6 images from color jittering, and 30 and 94 images from crop ratios of 8% and 12%, respectively, we skillfully expanded the initial dataset into a comprehensive collection comprising 360 images, denoted as Dataset-360. These augmented datasets serve as the foundational cornerstone for our thorough exploration of Barrett’s esophagus image segmentation using advanced deep-learning methodologies.

### 2.3. The Implementation Platform

We imported models from keras-unet-collection by Sha, Yingkai [10] to explore the Unet variants with available pretrained backbones and made a comparison with the Deeplabv3+ model based on the metric of meanIoU (intersection over union). Further, we conducted thorough ensemble learning across models and backbones. The keras-unet-collection has been enrolled in the Keras libraries and continuously maintained on the website https://github.com (accessed on 29 October 2023). We defined the hyper-parameters as follows:

model = models.unet_2d((128, 128, 3), filter_num = [64, 128, 256, 512, 1024];
n_labels = 1;
stack_num_down = 2, stack_num_up = 2;
activation = ‘ReLU’;
output_activation = ‘Sigmoid’;
batch_norm = True, pool = False, unpool = False;
backbone = ‘DenseNet121’, weights = ‘imagenet’;
freeze_backbone = True, freeze_batch_norm = True;
name = ‘unet’)

We first set the following hyperparameters: an input size or dimension (as 128, 128, 3), the convolutional filters (per down- and up-sampling blocks) and depth, n_labels = 1 (Binary), 2 convolutional layers per down sampling level, 2 convolutional layers (after concatenation) per up sampling level, activation of hidden layers = ‘ReLU’, output activation = ‘Sigmoid’, the configuration of down sampling and up sampling set as 2-by-2 convolution kernels with 2 strides. Usually, we adopted the default value except for VGG groups not using batch normalization, and we also set backbone parameter of those models from scratch to none. The details are available from the python helper function to run in models. Then, we compiled the program as follows:

model.compile(loss = ‘binary_crossentropy’, optimizer = Adam(learning_rate = 1 × 10^−5^),
metrics = [‘accuracy’, losses.dice_coef])

After adding a well-known model Deeplabv3+, for comparison, we also selected some models that obtained higher meanIoU to carry out further ensemble via grid search. The details will be described in the following subsection.

#### 2.3.1. Model and Backbone Network

The approach commences by choosing a variety of robust segmentation architectures, including U-Net [5], U-Net++ [11], Attention U-Net [12], and Recurrent Residual U-Net [13], derived from the keras-unet-collection by Sha, Yingkai [10]. The U-Net++ extends the U-Net architecture by introducing a series of nested and densely connected pathways, with each level supplying multi-scale contextual information to enhance segmentation accuracy. The Attention U-Net here is the variant that integrates attention gates into the U-Net architecture, allowing the dynamic modulation of information flow within the network. By assigning different weights to different spatial locations, attention gates enable the model to selectively focus on relevant regions while suppressing irrelevant features. The Recurrent Residual U-Net (R2U-Net) architecture incorporates recurrent convolutional layers and residual units. These architectural choices serve as the cornerstone of our segmentation framework. To achieve optimal performance, we conduct a thorough parameter fine-tuning process for each model, leveraging their inherent flexibility and configurability. In our endeavor to enhance these segmentation architectures, we rigorously investigate a variety of techniques and tools. These include altering the choice of backbone networks, with options spanning VGG16, VGG19 [13], ResNet50, ResNet101, ResNet152 [7], ResNet50V2, ResNet101V2, ResNet152V2 [14,15], DenseNet121, DenseNet169, and DenseNet201 [16]. The selection of a proper backbone is critical, as it directly affects the model’s ability to extract features and comprehend complex patterns within medical imaging data. Through this rigorous evaluation process, we aim to find and tailor segmentation models that are best suited for our specific task of Barrett’s esophagus segmentation. The comprehensive exploration and adaptation of well-established architectures constitute the fundamental groundwork for our subsequent investigations. These investigations encompass the integration and comparative analysis of the Deeplabv3+ [17] architecture, aimed at augmenting our segmentation strategy further.

#### 2.3.2. Model Training and Testing Process

The augmented datasets of 192 and 360 images were used for training on our platform. The datasets were resized to (128, 128) dimensions as the input image size, and we employed a batch size of 4 to accommodate the RTX3070 GPU. During model training, in the step of augmentation, we used the data generator configuration encompassing up to 15 degrees of rotation to encourage viewpoint invariance and a zoom range of up to 5% to further diversify the training set. We also augmented dataset-192 through both horizontal and vertical flips, with width and height shift ranges set to 0. During model training on dataset-360, the data generator configuration was same as mentioned above for dataset-192, except flips were disabled to keep the orientation of dataset-360.

**Figure 4:**
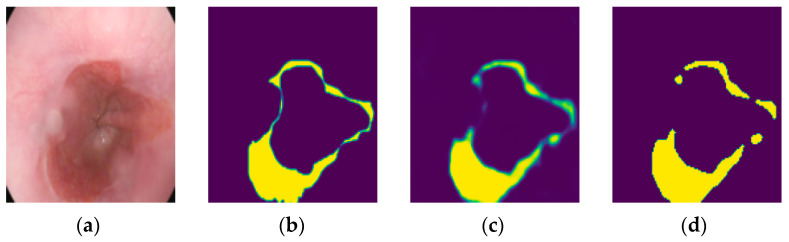
Post-processing of prediction: (**a**) Test image. (**b**) Ground truth mask. (**c**) Prediction mask before post-processing. (**d**) Prediction mask after post-processing.

For post-processing, we implemented a two-step approach involving thresholding and morphological operations on the predicted mask. We employed Otsu’s method to determine an optimal threshold for converting probability values into a binary mask. These operations are helpful in refining the binary mask, thereby enhancing the segmentation quality. Figure 4 presents a test image along with its corresponding ground truth mask, the prediction mask before post-processing, and the prediction mask after post-processing. However, we did not apply the test time augmentation due to our adequate 2-step data augmentation and the special dataset to be used.

**Figure 5:**
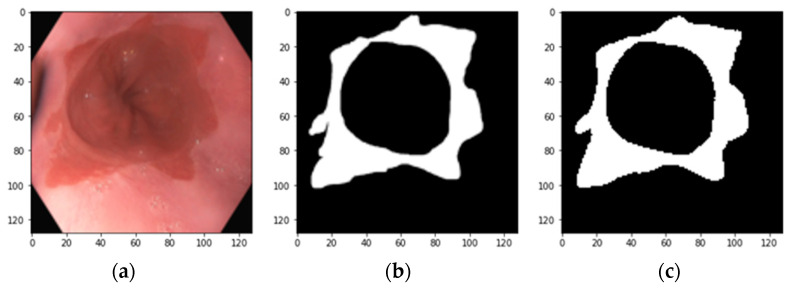
A testing image with its mask and prediction: (**a**) Test image. (**b**) Ground truth mask. (**c**) Prediction on the test image.

With the first learning rate of 1 × 10^−3^, after trial and error, our model was trained with a learning rate of 1 × 10^−5^ for a total of 120 epochs for comparison of models selected. During the training process, we implemented an early stopping criterion with a patience level of 20 and employed model checkpointing to track and save the model’s progress. To evaluate the performance of our model, we employed two key metrics: the dice coefficient and the mean intersection over union (meanIoU). We also recorded F1-Score for the test dataset. Figure 5 shows an original image, its ground truth, and prediction in order from left to right after the model training. We also ran more epochs for models gaining higher meanIoU to obtain the saturation of feature extraction.

### 2.4. Model Ensemble

**Figure 6:**
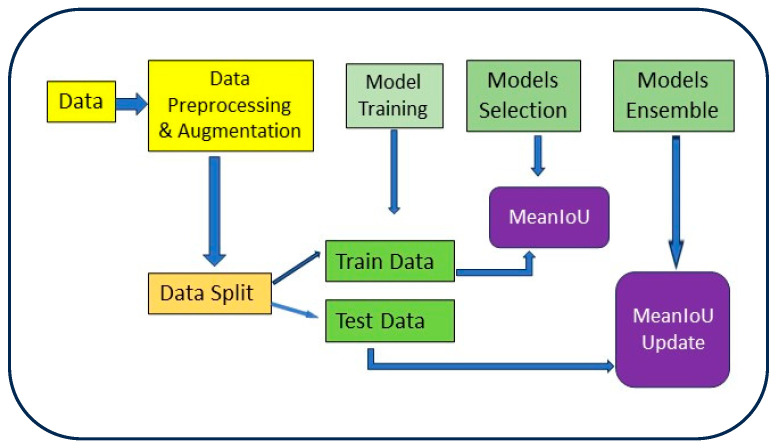
Workflow of model training and ensemble training.

We adopt a comprehensive ensemble strategy that includes all the available backbone configurations for each individual model. To improve the segmentation accuracy of Barrett’s esophagus, we explored the concept of ensemble learning, which combines the predictions of multiple models to create a more exact final prediction. To ensure reliable predictions, we set a prediction threshold of 0.5 for our ensemble model. This means that any pixel predicted with a probability equal to or higher than 0.5 is considered as Barrett’s esophagus class, while pixels with probabilities below 0.5 are classified as non-Barrett’s esophagus class. This approach ensures a thorough exploration of ensemble synergies across the entire architectural spectrum. To optimize the performance of our ensemble, we conducted an extensive grid search. This iterative process identifies the most effective weight ratios for combining predictions from the highest-performing models, determined based on their meanIoU scores. The workflow chart outlining this task is presented in Figure 6.

## 3. Results

### 3.1. Individual Model Performance

In the dataset-192, U-Net with a DenseNet121 backbone and attention U-Net with DenseNet169 exhibited the highest meanIoU scores of 0.85, both outperforming the deeplabv3+ that obtained the highest meanIoU of 0.78 with the DenseNet169, as shown in Table 1. Similarly, for the dataset-360, U-Net with a VGG16 backbone and deeplabv3+ with DensNet169, as shown in Table 2, achieve the highest meanIoU 0.90. It is noted that the Dense Net backbone consistently exhibited superior performance compared to other backbones across various U-Net variants, while Deeplabv3+ consistently performed well on both datasets. In Table 1 and Table 2, the empty cells indicate instances where the backbones failed to achieve a meanIoU score.

### 3.2. Ensemble Performance

The ensemble approach improved meanIoU in the dataset-360, demonstrating the synergistic strength of combining models. In the case of the dataset-192, the ensemble methods yielded more substantial improvements, resulting in an impressive meanIoU score increase of about 0.03. We noted that ResNet models might have adverse effects on models’ hybrid ensemble or backbone-based ensemble across all backbones enrolled.

### 3.3. Backbone-Based Ensemble Comparison

The ensemble that combines U-Net with various diverse backbones significantly increased meanIoU to 0.87 on the dataset-192. Moreover, on dataset-360, this ensemble consistently outperformed most individual models, underscoring its robustness and efficacy when dealing with diverse datasets. We observe that in dataset-360, the increase in meanIoU is smaller than that in dataset-192. This may be because the image features of dataset-360 are more fully extracted than those in dataset-192.

### 3.4. Comparison with Deeplabv3+

The comparison with Deeplabv3+ reveals the superiority of our approach on dataset-192, where our best ensemble outperformed Deeplabv3+. Remarkably, on dataset-360, the meanIoU values of our best ensemble matched those of Deeplabv3+, providing further validation of the effectiveness of our approach. Detailed comparisons between U-Net and Deeplabv3+ with the VGG16 backbone are presented in Table 3 and Table 4.

### 3.5. Grid Search Ensemble

The grid search ensemble, comprising models from three distinct backbone families, i.e., VGG16, ResNet50V2, and DenseNet121, achieved an optimal meanIoU of 0.86 on dataset-192. This outcome underscores its potential for fine-tuning model combinations. The models in this ensemble were based on U-Net with backbones from groups such as VGG16, ResNet101V2, and DensNet121, as outlined in Table 5 and Table 6. Similarly, significant success was observed on dataset-360, where the grid search ensemble contributed to the same meanIoU score of 0.91 for both the average ensemble and weighted ensemble with a weight ratio of 0.3, 0.2, and 0.3, as shown in Table 6. An ensemble of Deeplab3+ with three backbones as VGG16, ResNet50, and DenseNet201 obtained an average ensemble meanIoU of 0.90 and a weighted ensemble meanIoU of 0.91 but with a weight ratio of 0.4, 0.0, and 0.4, as shown in Table 6. Such results seem to mean that ResNet50 does not contribute to this task or is even harmful.

### 3.6. Model Ensemble from Scratch

To provide a comprehensive comparison, we evaluated several models that did not utilize a pretrained backbone in contrast to those with pretrained models. As depicted in Table 7 and Table 8, the ensemble of models consistently exhibited superior meanIoU compared to individual models, underscoring the benefits of model ensembling.

### 3.7. Saturation of Learning Rate and Ensemble Learning via Grid Search

From the models listed in Table 2, we chose seven models to undergo the training process with the previously mentioned settings. The training continued until the early stopping criterion was met, which allowed us to identify the saturation epochs for feature extraction. This process helped determine the optimal point at which feature extraction was most effective for our segmentation task. The models selected are listed as follows:

- Model 1: Unet with a backbone VGG16;
- Model 2: Unet with a backbone DenseNet121;
- Model 3: Attention Unet with a backbone DenseNet121;
- Model 4: deeplabv3+ with a backbone VGG16;
- Model 5: deeplabv3+ with a backbone VGG19;
- Model 6: deeplabv3+with a backbone DenseNet169;
- Model 7: deeplabv3+ with a backbone DenseNet201.

**Figure 7:**
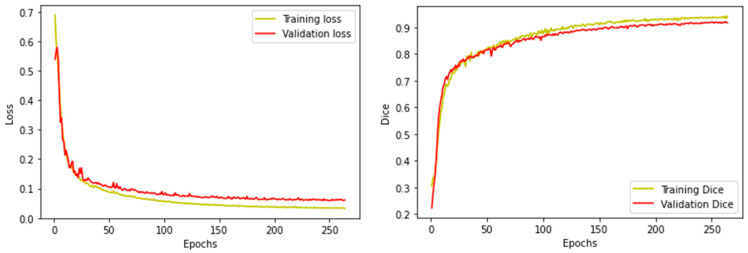
The training curve of Unet with a backbone VGG16.

The training loss and dice curves reaching the learning rate saturation of model 1 are shown in Figure 7, with a training loss of 0.0325, training accuracy of 0.9334, training dice coefficient of 0.9409, validation accuracy of 0.9391, validation dice coefficient of 0.9166.

In Table 9, the advanced meanIoU scores, as well as the corresponding epochs at which the selected models reached training saturation, are documented. These values provide insights into the model training process and help understand the point at which the models achieved optimal segmentation performance. It is a critical aspect of model development and validation in the context of Barrett’s esophagus segmentation. Then, we conducted a grid search to explore different combinations of two and three models among the seven selected models. These combinations were used in a thresholding soft voting ensemble learning approach. For the three-model combination, the best average ensemble meanIoU score of 0.918 was attained using the U-Net with a VGG16 backbone, Attention U-Net with a DenseNet121 backbone, and Deeplabv3+ with a DenseNet169 backbone, as listed in Table 10. The best average ensemble meanIoU score of 0.917 was achieved using a combination of two models, specifically the U-Net with a VGG16 backbone and Deeplabv3+ with a DenseNet169 backbone, as presented in Table 11. Furthermore, by employing weighted ensemble techniques for these model combinations, we obtained a meanIoU of 0.936, combining the U-Net with a VGG16 backbone and Deeplabv3+ with a DenseNet169 backbone, as illustrated in Table 12. These results demonstrate the effectiveness of our ensemble strategies in achieving high meanIoU scores, further enhancing the segmentation accuracy for Barrett’s esophagus.

## 4. Discussion

Pan et al. [8] first applied fully convolutional neural networks to perform Barrett’s esophagus segmentation. Their work inspired us to expand Barrett’s esophagus segmentation from severe esophageal cancer to early Barrett’s esophagus segmentation, which is also in line with early diagnosis and preliminary treatment of preventive medicine. In this study, we embarked on a comprehensive exploration of Barrett’s esophagus segmentation using deep-learning techniques. Through a rigorous method encompassing a diverse range of U-Net variants, extensive backbone selections, data augmentation strategies, and ensemble techniques, we aimed to achieve exact and robust segmentation results. The following key findings appear from our research.

### 4.1. Model Selection and Performance

Our investigation into various U-Net variants with different backbones revealed distinct performance patterns. In particular, U-Net with a DenseNet121 backbone and attention U-Net with DenseNet169 exhibited superior segmentation accuracy on the dataset-192, while on the more extensive dataset-360, U-Net with a VGG16 backbone showed a potential ability in this specific Barrett’s esophagus segmentation task. DenseNet models showed impressive segmentation accuracy, while ResNet models proved less effective for this task. DenseNet models use the famous dense connectivity pattern that enhances information flow throughout the network and promotes feature reuse, making it particularly well-suited for scenarios where capturing fine-grained details and exact segmentation boundaries are crucial. Nabil Ibtehaz and M. Sohel Rahman [18] made modifications to U-Net using their designed MultiRes Block and the residual path-like dense connectivity pattern. Their study called MultiResUNet attained a much higher Jaccard Index, a metric like IoU, than U-Net on diverse medical datasets. Their excellent study showed room for improvement of ResNet at specific tasks consistent with our research.

### 4.2. Impact of Data Augmentation

The application of data augmentation techniques played a pivotal role in enhancing model robustness and generalization. It is noted that augmenting the dataset-360 with added cropping, affine transformations, and color jittering leads to improved segmentation results, highlighting the importance of tailored augmentation strategies. However, we just applied affine transformations, color jittering, and other data augmentation techniques to about 25% of the dataset-360 except cropping. Cropping forms the majority (75%) of the augmentation in dataset-360. Thus, the core structure of the images is still relatively consistent, especially their spatial dimensions. Consistency can be important for medical image segmentation tasks to ensure that the model learns the fundamental anatomical features. On the other hand, the remaining 25% of augmentation is composed of affine transformations, color jittering, Gaussian blur, and noise. These techniques introduce variability, which can help the model to fit diverse real-world scenarios. While this percentage may seem small, it still contributes to diversifying the training data. The balance between consistency and variability is crucial for trading off between model overfitting and generalization. In this study, we adopted the limited rotation and zoom range for data augmentation and different flip choices between dataset-192 and dataset-360. In medical imaging, it is often essential to maintain anatomical correctness. Therefore, to keep consistency while introducing some variability using techniques like affine transformations, color jittering, and noise is a reasonable approach. The chosen balance is effective in this context. Data augmentation can be helpful for improving the performance of deep-learning models on medical image datasets; however, it cannot inherently increase the amount of data available, like novel data acquisition.

### 4.3. Models with vs. without a Backbone

Models utilizing specific backbones, such as those from the VGG and DenseNet groups, achieved higher meanIoU scores. Nevertheless, it is noteworthy that a U-Net model trained from scratch managed to attain a commendable meanIoU of 0.88 on dataset-360, underscoring its performance in this context.

### 4.4. Ensemble Strategies

Our ensemble strategies have demonstrated their potential to achieve superior segmentation accuracy. The ensemble of U-Net models on dataset-360 achieves a remarkable meanIoU score of 0.91, confirming the efficacy of combining diverse variants for enhanced performance. The fine-tuning of ensemble weights through grid search further contributes to the improved results. It is noted an average ensemble of Vgg16, ResNet50, and DenseNet201 in Deeplabv3+ obtained a meanIoU of 0.90, while a weighted ensemble with ratios 0.4, 0.0, and 0.4 obtained 0.91. This aligns with the observations in our study, where ResNet variants exhibited mixed results. In particular, ResNet50V2 and ResNet101V2 demonstrated competitive segmentation accuracy, while other variants showed limitations in managing the increased dataset complexity.

### 4.5. Comparison with Deeplabv3

Through a comprehensive comparison with the popular Deeplabv3+ architecture, we highlighted the influence of backbone choice on segmentation performance. While the U-Net variants with specific backbones outperformed Deeplabv3+ on dataset-192, the importance of aligning models to data characteristics became evident. Intriguingly, we observed that some backbone combinations struggle to achieve satisfactory results on dataset-192, showing the impact of data characteristics on model training. Extending the comparison to dataset-360, we observed similar best meanIoU scores among U-Net variants and Deeplabv3+, reaching a noteworthy score of 0.91. This observation underscores the adaptability of our approach to larger datasets, facilitating robust and accurate Barrett’s esophagus segmentation. The integration of transfer learning, data augmentation, and ensemble techniques collectively contributed to surpassing the performance of a well-established architecture such as Deeplabv3+.

### 4.6. Training Saturation and Ensemble Learning

The selected seven models demonstrated high meanIoU scores, indicating their effectiveness in segmenting Barrett’s esophagus. All models reached training saturation, and the number of epochs required for this varied across models. This information can be used to optimize training resources and time for future experiments. Models with fewer epochs might be preferable due to efficiency. The combination of models 1, 3, and 6 in Table 9 achieved a weighted ensemble meanIoU of 0.936. The weight ratio used for this combination was [0.4, 0.0, 0.2]. Like the previous combination, the ensemble consisting of models 1 and 6 also achieved a weighted ensemble meanIoU of 0.936 with the weight ratio [0.4, 0.2]. This means that in the ensemble, model 1 contributed 40% to the final prediction, model 3 had no contribution (0%), and model 6 contributed 20%. This result indicates that a simpler ensemble of two models can achieve the same high meanIoU score as the more complex three-model combination.

**Figure 8:**
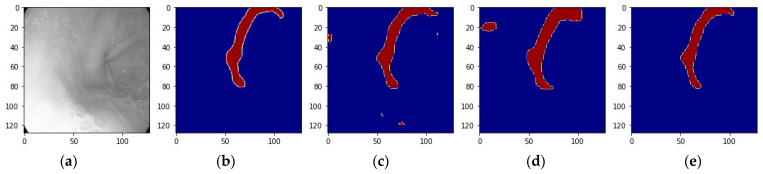
The ensemble collaboration of model 1 and model 6: (**a**) The example image in grey, (**b**) ground truth of the image, (**c**) Prediction of model 1, (**d**) Prediction of model 6, (**e**) The prediction of ensemble learning of model 1 and model 6.

**Figure 9:**
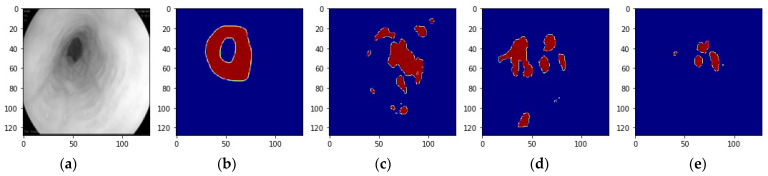
The ensemble collaboration of model 1 and model 6: (**a**) Another example image in grey, (**b**) Ground truth of the image, (**c**) Prediction of model 1, (**d**) Prediction of model 6, (**e**) The prediction of ensemble learning of model 1 and model 6.

To illustrate the transparency and interpretability of artificial intelligence, we visualized the predictions from ensemble learning of model 1 and model 6. Figure 8e presents the ensemble prediction of image Figure 8a. It removed the redundancy of predictions of model 1 and model 6, noted as Figure 8c and Figure 8d, and obtained a prediction closer to the ground truth noted as Figure 8b. However, in Figure 9, the predictions of model 1 and model 6 noted as Figure 9c and Figure 9d were limited due to the uneven heterogeneity of image Figure 9a compared to the ground truth noted as Figure 9b. The ensemble prediction Figure 9e showed that the annotation of the image was difficult to perfect and pointed out the directions still to be worked on in the future.

## 5. Conclusions

In this comprehensive study on Barrett’s esophagus segmentation, we have explored the effectiveness of deep-learning techniques, model variations, data augmentation, and ensemble strategies to achieve precise and robust results. Our findings provide essential insights and draw significant conclusions. Obviously, the choice of model variants and backbones significantly impacts segmentation accuracy. Models with DenseNet backbones demonstrated superior performance, attributed to their dense connectivity pattern, which enhances feature reuse and information flow. Tailored data augmentation strategies, combining spatial consistency with variability, proved crucial for enhancing model generalization. They strike a balance between consistency, crucial for maintaining anatomical correctness, and variability, essential for real-world scenarios. Models with specific backbones, particularly from the VGG and DenseNet groups, consistently achieved higher meanIoU scores. Surprisingly, a U-Net model trained from scratch showed commendable performance, indicating its potential in Barrett’s esophagus segmentation. The ensemble of U-Net models resulted in improved segmentation accuracy, and the finetuning of ensemble weights via grid search further enhanced the segmentation results. This ensemble strategy effectively leveraged the strengths of different U-Net models to create a more accurate and robust final segmentation output. It highlights the significance of ensemble learning in achieving superior segmentation performance. These findings underscore the effectiveness of combining diverse model variants.

Our approach has demonstrated significant improvements in Barrett’s esophagus segmentation, and it has outperformed the Deeplabv3+ architecture on the dataset-192. This highlights the importance of selecting the right models that match the data characteristics for optimal performance. On the larger dataset-360, both U-Net variants and Deeplabv3+ achieved high meanIoU scores, showcasing the adaptability of our approach to larger datasets. Our research also provides insights into the training process, indicating that models reach training saturation at varying epochs, which can help optimize training resources. Additionally, our findings show that combining models with fewer epochs in simpler ensembles can achieve similar high meanIoU scores as more complex combinations, emphasizing the efficiency of simpler approaches. The visible predictions of ensemble models have emphasized the importance of high-quality annotations in segmentation tasks, and our approach represents a significant advancement in Barrett’s esophagus segmentation, facilitating precise and reliable medical image analysis. The ensemble strategies, combined with data augmentation and model collaboration, offer a robust solution. These insights have the potential to contribute to further advancements in medical image analysis.

## Figures and Tables

**Table 1:** Ensemble MeanIoU of models with backbones (Dataset-192).

Model Backbone	Unet	Unet2plus	Attension Unet	Deeplab v3+	Ensemble
VGG16	0.81	0.51	0.80	0.77	0.81
VGG19	0.80	0.46	0.78	0.76	0.81
ResNet50	0.56	0.42	0.58		0.52
ResNet101	0.53	0.42	0.53		050
ResNet152	0.59	0.42	0.58		0.55
ResNet50V2	0.83	0.81	0.83		0.85
ResNet101V2	0.83	0.78	0.80		0.83
ResNet152V2	0.83	0.77	0.80		0.83
DenseNet121	0.85	0.83	0.84		0.86
DenseNet169	0.84	0.84	0.85	0.78	0.86
DenseNet201	0.83	0.83	0.83	0.78	0.85
Ensemble	0.87	0.76	0.80	0.80	

**Table 2:** Ensemble MeanIoU of models with backbones (Dataset-360).

Model Backbone	Unet	Unet 2plus	Attension Unet	Deeplab v3+	Ensemble
VGG16	0.90	0.75	0.88	0.89	0.90
VGG19	0.88	0.73	0.84	0.90	0.89
ResNet50	0.65	0.47	0.68	0.79	0.77
ResNet101	0.69	0.51	0.69	0.76	0.73
ResNet152	0.71	0.44	0.62		0.71
ResNet50V2	0.86	0.84	0.87		0.88
ResNet101V2	0.87	0.82	0.86		0.88
ResNet152 V2	0.83	0.76	0.84		0.84
DenseNet121	0.88	0.87	0.88		0.89
DenseNet169	0.86	0.86	0.87	0.90	0.89
DenseNet201	0.88	0.86	0.88	0.90	0.90
Ensemble	0.90	0.87	0.90	0.90	

**Table 3:** Comparison of UNet and Deeplabv3+ with backbone VGG16 (Dataset-192).

Metrics	IoU	Dice	F1-Score
UNet	0.82	0.76	0.82
Deeplabv3+	0.77	0.68	0.78

**Table 4:** Comparison of UNet and Deeplabv3+ with backbone VGG16 (Dataset-360).

Metrics	IoU	Dice	F1-Score
UNet	0.90	0.87	0.91
Deeplabv3+	0.89	0.87	0.91

**Table 5:** Ensemble MeanIoU of each model with three backbones (Dataset-192).

Model	Backbones	Average Ensemble
Unet	VGG16 ResNet50V2 DenseNet121	0.86
Unet 2plus	VGG16 ResNet50V2 DenseNet169	0.84
Attension Unet	VGG16 ResNet50V2 DenseNet169	0.84
Deeplabv3+	VGG16, DenseNet169 DenseNet201	0.80

**Table 6:** Ensemble MeanIoU of each model with three backbones (Dataset-360).

Model	Backbones	Average Ensemble	Weighted Ensemble	Weighted Ratio
Unet	VGG16 ResNet101V2 DenseNet121	0.91	0.91	[0.3, 0.2, 0.3]
Unet 2plus	VGG16 ResNet50V2 DenseNet121	0.87	0.88	[0.1, 0.3, 0.4]
Attension Unet	VGG16 ResNet50V2 DenseNet121	0.90	0.90	[0.4, 0.1, 0.3]
Deeplabv3+	VGG16 ResNet50 DenseNet201	0.90	0.91	[0.4,0.0, 0.4]

**Table 7:** Ensemble MeanIoU of variants of Unet without a backbone (Dataset-192).

Model	Unet	Unet2plus	Attention Unet	R2Unet	Ensemble
MeanIoU	0.80	0.80	0.82	0.78	0.83

**Table 8:** Ensemble MeanIoU of variants of Unet without a backbone (Dataset-360).

Model	Unet	Unet2plus	Attention Unet	R2Unet	Ensemble
MeanIoU	0.88	0.82	0.84	0.87	0.89

**Table 9:** The advanced meanIoU and the epochs reaching training saturation.

Models	meanIoU	Epochs
Model 1	0.90	264
Model 2	0.89	175
Model 3	0.89	153
Model 4	0.90	264
Model 5	0.90	249
Model 6	0.91	306
Model 7	0.90	202

**Table 10:** Top 6 average ensemble meanIoU of 3 model combinations.

Model Combinations	Average Ensemble meanIoU
[model 1, model 3, model 6]	0.918
[model 3, model 5, model 6]	0.917
[model 1, model 5, model 6]	0.917
[model 1, model 6, model 7]	0.917
[model 1, model 4, model 6]	0.916
[model 1, model 3, model 5]	0.916

**Table 11:** Top 6 average ensemble meanIoU of 2 model combinations.

Model Combinations	Average Ensemble meanIoU
[model 1, model 6]	0.917
[model 1, model 7]	0.914
[model 3, model 6]	0.914
[model 5, model 6]	0.913
[model 4, model 6]	0.913
[model 1, model 5]	0.913

**Table 12:** Best weighted ensemble meanIoU of 2 and 3 model combinations.

Model Combinations	Weighted Ensemble meanIoU	Weight Ratio
[model 1, model 3, model 6]	0.936	[0.4, 0.0, 0.2]
[model 1, model 6]	0.936	[0.4, 0.2]

## Data Availability

There is no new data generated or analyzed in support of this research.
